# Formation of nano-sized M_2_C carbides in Si-free GH3535 alloy

**DOI:** 10.1038/s41598-018-26426-0

**Published:** 2018-05-25

**Authors:** Li Jiang, Wang Yinling, Rui Hu, Renduo Liu, Xiang-Xi Ye, Zhijun Li, Xingtai Zhou

**Affiliations:** 1 Shanghai Institute of Applied Physics, Chinese Academy of Sciences, Shanghai, 201800 P.R. China; 2 College of Mechanical and Energy Engineering, Huanghuai University, Zhumadian, 463000 P.R. China; 3 State Key Laboratory of Solidification Processing, Northwestern Polytechnical University, Xi’an, 710072 P.R. China

## Abstract

GH3535 alloy is one of the most promising structural materials for molten salt reactors (MSRs). Its microstructure is characterized by equiaxed grains and coarser primary M_6_C carbide strings. In this study, stable nano-sized M_2_C carbides were obtained in GH3535 alloy by the removal of Si and thermal exposure at 650 °C. Nano-sized M_2_C carbide particles precipitate preferentially at grain boundaries during the initial stage of thermal exposure and then spread all over the grain interior in two forms, namely, arrays along the {1 1 1} planes and randomly distributed particles. The precipitate-free zones (PFZs) and the precipitate-enriched zones (PEZs) of the M_2_C carbides were found to coexist in the vicinity of the grain boundaries. All M_2_C carbides possess one certain orientation relationship (OR) with the matrix. Based on microstructural characterizations, the formation process of M_2_C carbides with different morphologies was discussed. The results suggested that the more-stable morphology and OR of M_2_C carbides in the Si-free alloy provide higher hardness and better post-irradiation properties, as reported previously. Our results indicate the preferential application of Si-free GH3535 alloy for the low-temperature components in MSRs.

## Introduction

Hastelloy N alloy, also called GH3535 alloy in China, is a Ni-Mo-Cr-based superalloy for molten salt reactors (MSRs) that contains 17 wt.% molybdenum for strengthening and 7 wt.% chromium, which is sufficient to impart moderate oxidation resistance in air but not enough to lead to high corrosion rates in salt^1^. As a deoxidant, 0.5–1 wt.% Si is added to air-melted heats of this alloy, unlike in vacuum-melted heats.

Hastelloy N alloy has been used for all salt-contacting parts of MSR systems, including the core vessel, heat exchangers and piping, and the service temperatures of these parts range from 550 °C to 700 °C^2^. Researchers have paid considerable attention to the effects of long-term thermal exposure on the microstructures and properties of this alloy. The types and morphologies of secondary precipitates at the grain boundaries were found to strongly depend on the Si content. When the Si-free alloy was exposed to temperatures in the range from 700 °C to 800 °C, nano-sized M_2_C carbides precipitated preferentially at grain boundaries during the initial stage of thermal exposure and then completely transformed into massive, plate-like M_6_C carbides as the exposure time increased^3,4^. In the case of the Si-containing alloy (0.46 wt.%), granular M_6_C carbides formed at the grain boundaries and remained stable during the whole exposure time^5^. Massive, plate-like M_6_C carbides, which transformed from M_2_C carbides, have been reported to act as crack nucleation sites at the grain boundaries, and they more severely deteriorated the tensile and creep properties of the Si-free alloy at 700 °C than the Si-containing one (0.46 wt.%)^5,6^.

In contrast, Si-free alloys possess better mechanical properties after short-term thermal exposure, under which fine intergranular M_2_C carbides do not transform into massive M_6_C carbides. The tensile elongation of the Si-free alloy was reported to be higher than that of the Si-containing alloy after exposure to 700 °C for 100 hours^7^. The strengthening effect was attributed to only the precipitation of nano-sized grain-boundary M_2_C carbides. The transformation dynamics from M_2_C to M_6_C carbides has been observed to become sluggish when the thermal exposure temperature drops from 800 °C to 700 °C. Thus, whether fine M_2_C carbides can form and remain stable in the Si-free alloy at a lower temperature, for example, 650 °C, and improve the service performance, as mentioned above, remains unknown.

Although a higher outer temperature is desired in new MSR designs, many components of the MSR would still operate at 650 °C or lower. However, the microstructural characteristics of the M_2_C carbide itself during thermal exposure to this temperature range remain unclear. In this study, a Si-free GH3535 alloy was thermally exposed to 650 °C for up to 2000 hours to determine the microstructural evolution of M_2_C carbides. For comparison, a Si-containing alloy was also analysed in this study. The stable nano-sized M_2_C carbides were found to form not only at grain boundaries but also in the matrix after thermal exposure at 650 °C. The formation mechanism of these M_2_C carbides and their effects on the alloy performance were also discussed.

## Results

### Morphological evolution of grain-boundary M_6_C carbides in Si-containing alloy

**Figure 1:**
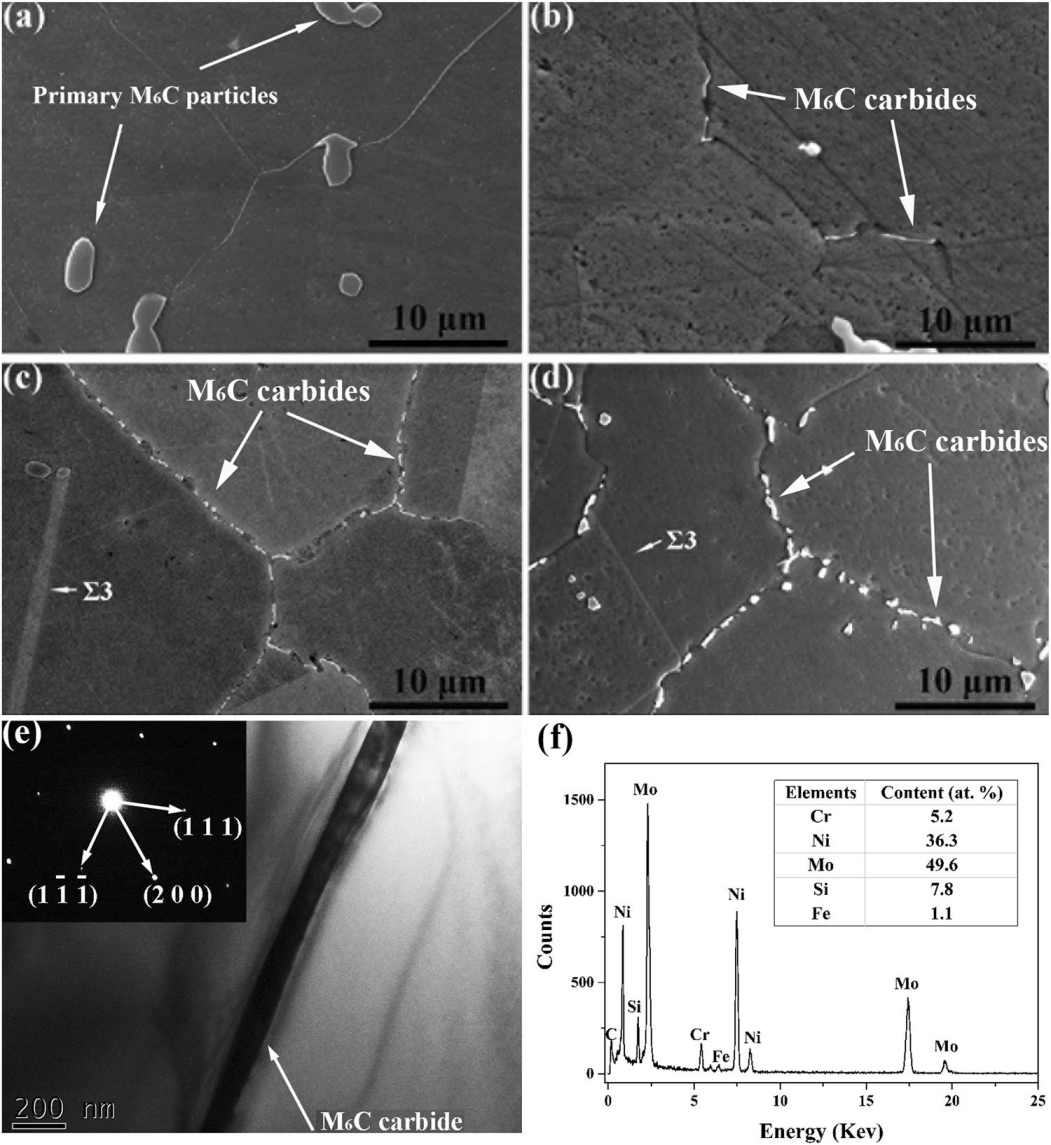
Morphologies of grain-boundary precipitates in the Si-containing alloy under different thermal exposure conditions. **(a)** Solution treatment, **(b)** 650 °C for 1 hour, **(c)** 650 °C for 500 hours, and **(d)** 650 °C for 2000 hours. **(e)** TEM image and corresponding SADP for M_6_C carbides in the Si-containing alloy exposed to 650 °C for one hour. **(f)** Typical TEM-EDX spectrums and semi-quantitative compositional analyses for the M_6_C carbides in the Si-containing alloy. The carbon content is difficult to detect accurately using EDX, so only the relative contents (at.%) of the metal atoms (Cr, Ni, Mo, Si and Fe) are listed.

For comparison, the morphological evolution of grain-boundary M_6_C carbides in the Si-containing alloy was investigated in this study. The microstructure is characterized by equiaxed grains and coarser primary M_6_C carbide strings along the rolling direction in the Si-containing alloy, as reported in our previous study^8^. These primary M_6_C carbide particles range in size from ~0.5 to 5 μm, as shown in Fig. 1a. No secondary precipitates can be observed at the grain boundaries in the solution treatment state (Fig. 1a). After one hour of exposure, a small number of precipitates can be observed at some of the grain boundaries (Fig. 1b), which were identified as M_6_C carbides using the select area diffraction pattern (SADP) from transmission electron microscopy (TEM) (Fig. 1e). As the exposure time increases to 500 hours, the M_6_C carbides continue to form and cover all grain boundaries except the twin ones, as shown in Fig. 1c,d. Except for the primary M_6_C carbide particles, no secondary precipitates can be observed in the grain interior after thermal exposure in the Si-containing alloy. The typical TEM-EDX spectrum and the semi-quantitative composition analyses are shown in Fig. 1f. The M_6_C carbides contain mainly Mo, Ni, Cr, Si and a small amount of Fe. In contrast with the surrounding matrix, the in M_6_C carbides are obviously richer in Mo and Si.

### Morphological evolution of M_2_C carbides in the Si-free alloy

**Figure 2:**
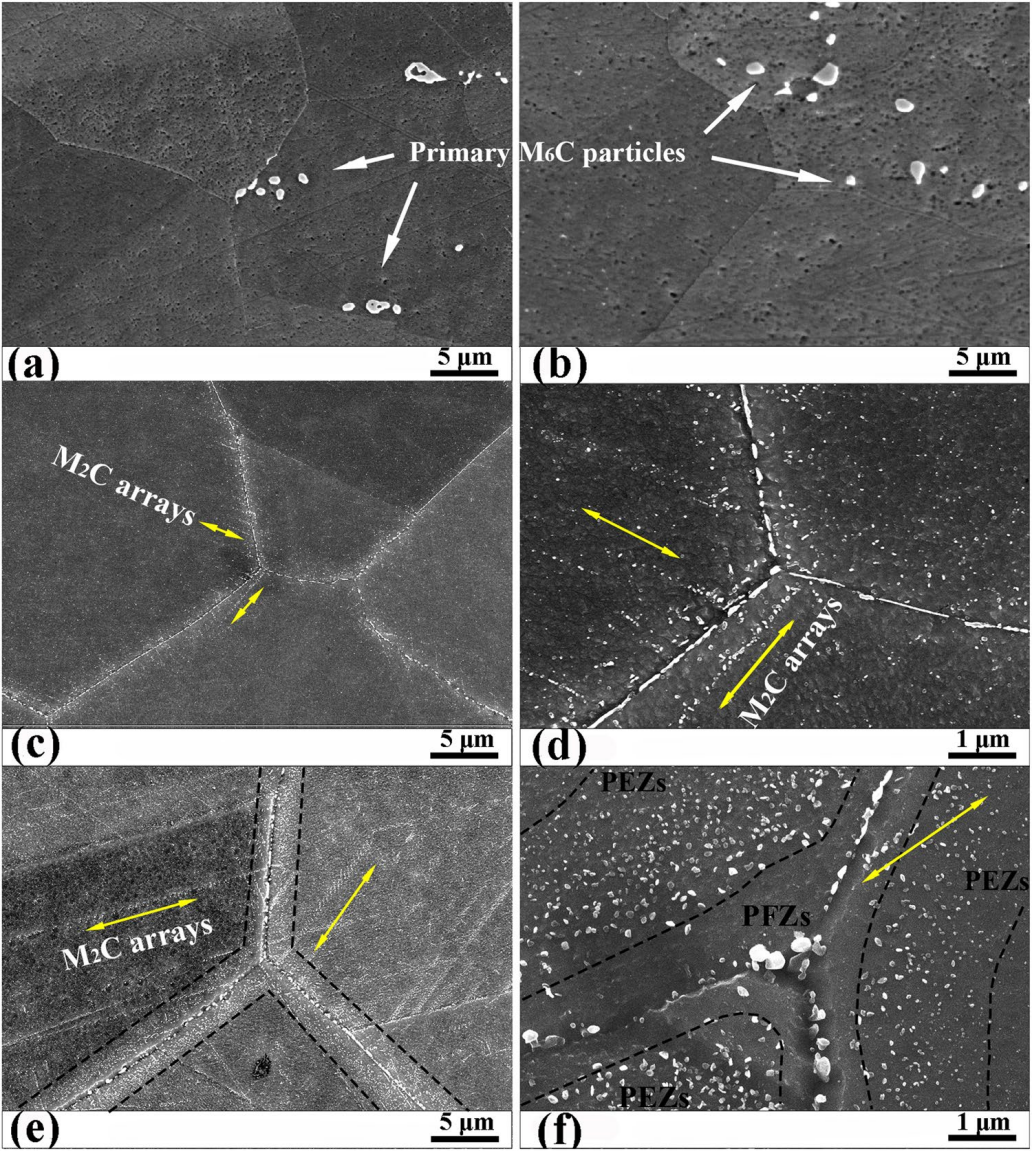
Morphologies of nano-sized precipitates in the Si-free alloy under different thermal exposure conditions. **(a)** Solution treatment, **(b)** 650 °C for 1 hour, **(c)** 650 °C for 500 hours, **(d)** the high-resolution image for **(c)**, **(e)** at 650 °C for 2000 hours, and **(f)** the high-resolution image for **(e)**. The yellow arrows in (**c**–**f**) indicate the certain direction of the precipitate arrays. In **(e,f)**, the PEZs and PFZs are enclosed by black dotted lines.

As shown in Fig. 2a, the microstructure of the Si-free alloy in the solution treatment state is similar to that of the Si-containing alloy. In fact, the M_2_C eutectic carbides and M_6_C eutectic carbides were detected in the as-cast Si-free GH3535 alloy^4^. When the as-cast alloy was rolled into a wrought alloy, the M_2_C eutectic carbides were crushed and completely decomposed during the subsequent solution treatment at 1177 °C, while the more stable M_6_C eutectic carbides were crushed into strings along the hot rolling direction and remained after this solution treatment^4^. After one hour of thermal exposure, the grain boundaries were still free of precipitates (Fig. 2b). As the exposure time increased to 500 hours, dense granular precipitates were found at all grain boundaries except the twin boundaries (Σ3 boundaries), as shown in Fig. 2c. In the high-resolution image (Fig. 2d), smaller granular precipitates were found to lie along a certain direction (as marked by yellow arrows) in the grain boundary regions with a width of ~4 μm, which we call precipitate arrays. With a longer thermal exposure of 2000 hours, the granular precipitates at the grain boundaries coarsen, and the precipitate arrays extend from the grain boundary regions into the whole grain interior (Fig. 2e). At the same time, the precipitate-free zones (PFZs) with a width of ~1 μm and the precipitate-enriched zones (PEZs) with a width of ~2 μm can be observed within the grain boundary region. Other than the preformed precipitate arrays (marked by yellow arrows in Fig. 2f), no precipitates can be observed in the PFZs. Surprisingly, a high density of precipitates (~34/μm^2^) are distributed in the adjacent PEZs.

**Figure 3:**
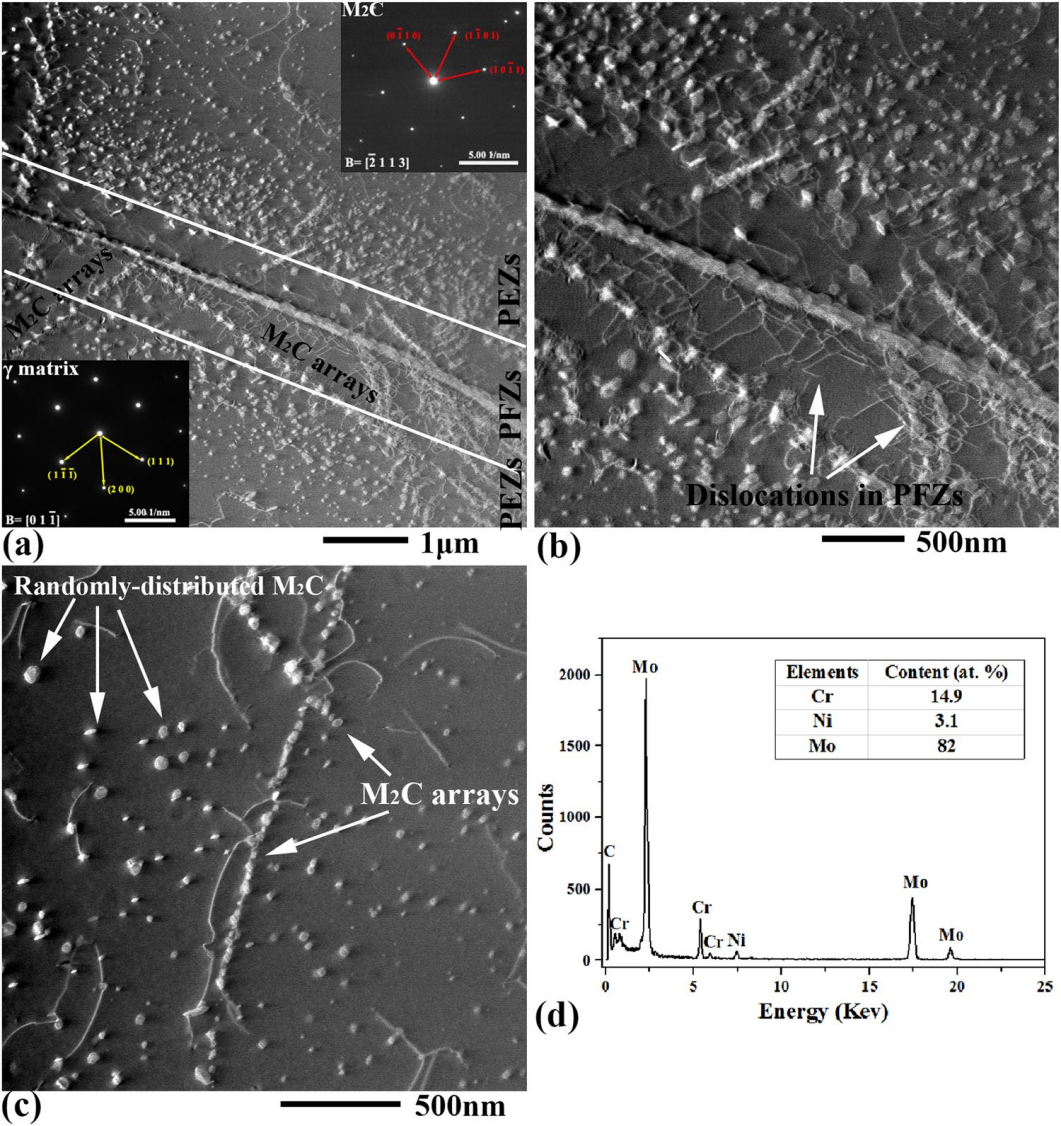
STEM image and corresponding SADPs for the M_2_C carbides in the Si-free alloy exposed to 650 °C for 2000 hours. **(a)** Within the grain boundary region (at grain boundaries or in the precipitate arrays and PEZs) and **(b)** the high-resolution image for **(a)**. **(c)** Within the matrix (randomly distributed or in arrays). **(d)** Typical TEM-EDX spectrums and semi-quantitative composition analyses for the M_2_C carbides in the Si-free alloy. The carbon content is difficult to detect accurately using EDX, so only the relative contents (at. %) of the metal atoms (Cr, Ni and Mo) are listed.

**Figure 4:**
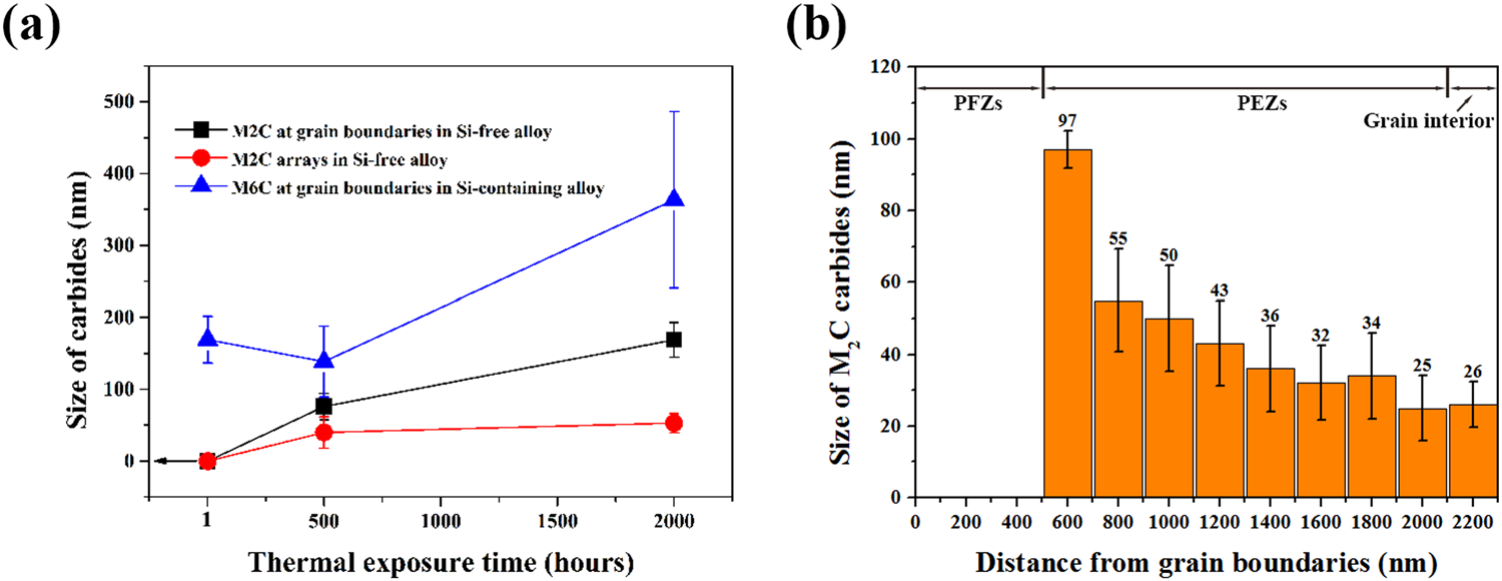
Size of precipitates in the Si-free and Si-containing alloys. **(a)** The size of the M_2_C carbides in the Si-free alloy and the M_6_C carbides in the Si-containing alloy as a function of the exposure time. **(b)** The size of the M_2_C carbides in the PEZs as a function of the distance from the grain boundaries.

Except for the coarser primary M_6_C carbides, nano-sized secondary precipitates in the different regions (grain boundaries, arrays and PEZs) were all identified as M_2_C-type carbides using TEM. Figure 3 shows the typical scanning TEM (STEM) images and corresponding SADP for the sample exposed for 2000 hours. The diffraction patterns of the M_2_C carbides could be indexed to a hexagonal structure with a = ~0.298 and c = ~0.476 nm (c/a = 1.59). This result agrees well with the values reported in the previous study^9^. Additionally, the STEM images can provide more details of the microstructures in the grain boundary regions (Fig. 3a,b) and the matrix (Fig. 3c) that cannot be revealed by scanning electron microscope (SEM) images (Fig. 2). As shown in Fig. 3a, two arrays of the M_2_C carbides pass completely through the PEZs and PFZs and intersect the grain boundary. The identical orientation of the two arrays and the {1 1 1} planes of the matrix indicates that the M_2_C carbides tend to grow along the close-packed plane of the matrix. On the other hand, dense dislocations are found to distribute in the PFZs and to connect the M_2_C carbides at the grain boundaries with the ones in the PEZs (Fig. 3b). Additionally, the M_2_C carbide particles in the arrays are connected to each other by dislocations. In the grain interior, the randomly distributed M_2_C carbide particles can be found to coexist with the arrays of M_2_C carbides (Fig. 3c). The density of the randomly distributed M_2_C carbide particles (~11/μm^2^) is far lower than that in the PEZs (~34/μm^2^), and their size (~26 nm) is shown in Fig. 4b. The typical TEM-EDX spectrum and the semi-quantitative compositional analyses are shown in Fig. 3d. The M_2_C carbides mainly contain Mo, Cr and a small number of metallic Ni atoms. The M_2_C carbides are obviously richer in Mo and Cr than the surrounding matrix.

To evaluate the coarsening kinetics, the sizes of the M_2_C carbides in the Si-free alloy and the M_6_C carbides in the Si-containing alloy were measured and compared, as shown in Fig. 4. When the exposure time increases from 500 hours to 2000 hours, the size of the M_2_C carbides in the arrays increases slightly from ~40 nm to ~53 nm (Fig. 4a). In contrast, the size of the grain-boundary M_2_C carbides more than doubled, increasing up to ~169 nm (Fig. 4a). The M_2_C carbides in the PEZs are characterized by a steep size gradient, decreasing from ~96 nm to ~26 nm with the increasing distance from the PFZs (Fig. 4b). In the Si-containing alloy, the grain-boundary M_6_C carbides exhibit stronger coarsening kinetics, increasing to ~360 nm after 2000 hour of thermal exposure.

### Orientation relationships

To examine the orientation relationship (OR) between the matrix and the grain-boundary carbides, the electron backscatter diffraction (EBSD) measurements were carried out in several regions, including the grain-boundary carbides and the two neighbouring grains that give essentially the same results. In the Si-containing alloy, no specific OR can be found between the grain-boundary M_6_C carbides and the matrix.

**Figure 5:**
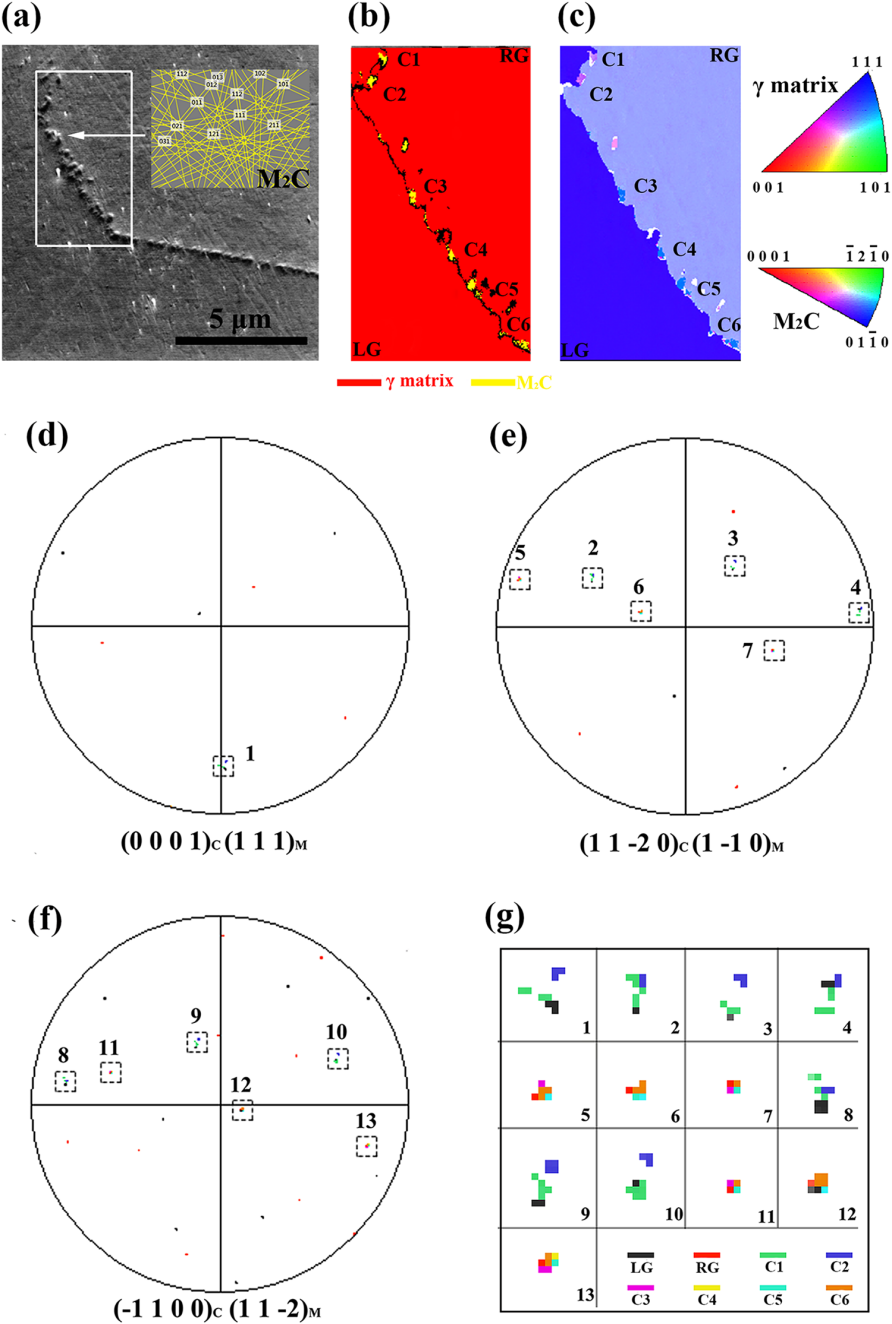
Orientation relationship between the M_2_C carbides and the matrix using EBSD. **(a)** M_2_C carbides at the grain boundaries in the sample exposed for 2000 hours and the typical Kikuchi patterns. EBSD phase map **(b)** and IPF map **(c)** for the M_2_C carbides and the γ matrix in the region marked by the white box in **(a)**. The colours in the IPF map **(c)** correspond to the crystallographic orientations indicated in the inverse pole figure. In **(b,c)**, the six grain-boundary M_2_C carbide particles were labelled “C1”, “C2”, “C3”, “C4”, “C5” and “C6”, and the two neighbouring grains were labelled “LG” and “RG” for the following discussions. **(d–f)** Show the pole figures for the {0 0 0 1}−, {1 1 −2 0} and {−1 1 0 0} planes in the grain-boundary M_2_C carbides (C1-C6), and the {1 1 1}, {1 −1 0} and {1 1 −2} planes in the two neighbouring grains (LG and RG). These spots with similar positions in the pole figures are numbered 1 to 13 and magnified as shown in **(g)**.

As shown in Fig. 5a, the grain-boundary precipitates are verified to be M_2_C carbides by the Kikuchi patterns in the Si-free alloy exposed for 2000 hours. From the EBSD phase map (Fig. 5b) and the inverse pole figure (IPF) map (Fig. 5c), six M_2_C carbide particles can be observed at the grain boundary, which were labelled C1, C2, C3, C4, C5 and C6. The two neighbouring grains were labelled LG and RG. The pole figures for the {0 0 0 1}, {1 1 −2 0} and {−1 1 0 0} planes were obtained from the six grain-boundary M_2_C carbide particles, and the ones for the {1 1 1}, {1 −1 0} and {1 1 −2} planes were obtained from the two neighbouring grains, as shown in Fig. 5d–f. The spots of the {0 0 0 1}, {1 1 −2 0} and {−1 1 0 0} planes in the M_2_C carbide particles C1 and C2 are near those of the {1 1 1}, {1 −1 0} and {1 1 −2} planes in LG, respectively. The same relation can also be observed between the M_2_C carbide particles C3-C6 and RG. Thus, the OR between the matrix γ phase and M_2_C carbides can be stated as: ${(0001)}_{c}//{(111)}_{\gamma }$, ${(11\bar{2}0)}_{c}//{(1\bar{1}0)}_{\gamma }$ and ${(\bar{1}100)}_{c}//{(11\bar{2})}_{\gamma }$.

Where *c* denotes the M_2_C carbides. The high-resolution image (Fig. 5g) shows a deviation from the strict OR. This OR was initially predicted by the assumptions of the Jack OR between the M_2_C carbides and the ferrite and the Kurdjumov-Sach (K-S) OR between the ferrite and the austenite in the steels^10^ and was finally confirmed by TEM in a Fe-Mn-Al-Mo-C alloy^11^. In our work, this OR was also verified using large-area and multi-zone EBSD measurements.

**Figure 6:**
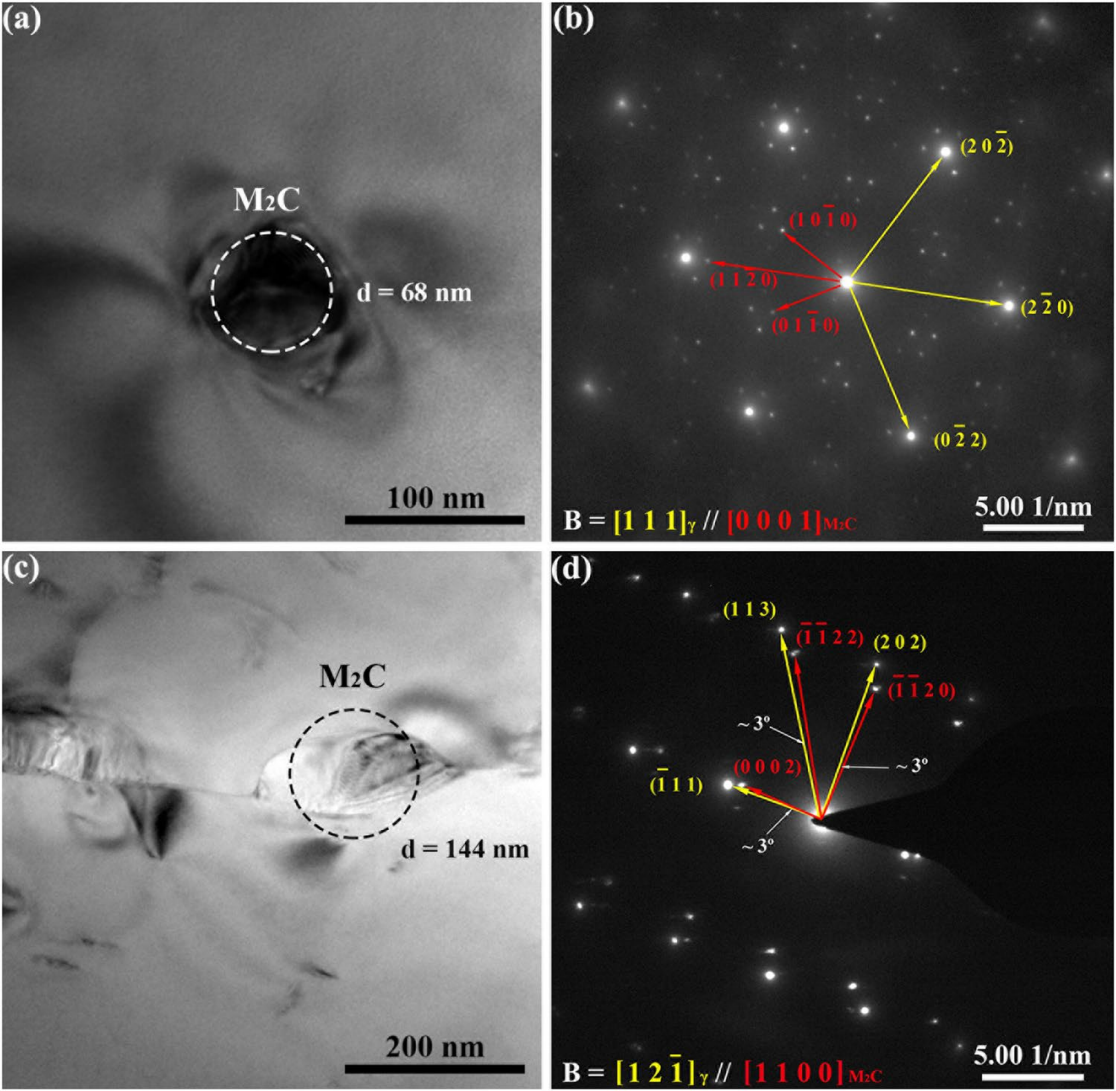
Orientation relationship between the M_2_C carbides and the matrix using TEM. **(a)** Bright-field TEM morphology for a M_2_C carbide particle in a PEZ with a size of ~68 nm. **(b)** SAED patterns for the M_2_C carbide particle in **(a)** and the surrounding matrix, the zone axis is [1 1 1] in the γ matrix and [0 0 0 1] in the M_2_C carbide particle. **(c)** Bright-field TEM morphology for an ~144 nm M_2_C carbide particle at the grain boundary. **(d)** SAED patterns for the M_2_C carbide particle in **(c)** and the surrounding matrix, the zone axes are [1 2 −1] in the γ matrix and [1 1 0 0] in the M_2_C carbide particle. In **(a,c)**, the circle possesses the same area as the carbide particle to indicate the size. In **(b,d)**, the yellow arrows and annotations indicate the γ matrix, and the red arrows indicate the M_2_C carbides.

The spatial and angular resolutions of EBSD are limited, as defined by the material interaction volume with the electron beam in a SEM. In our study, a spatial resolution of down to ~100 nm was achieved, which is inadequate to deal with the fine M_2_C carbides in arrays, PEZs and grain interiors. Thus, TEM was applied to investigate these particles smaller than 100 nm. The typical TEM morphologies and SAED patterns for the M_2_C carbide particles are shown in Fig. 6. According to the SAED patterns (Fig. 6b), the M_2_C carbide particle (~68 nm) exhibits the strict OR with the surrounding matrix, as revealed using EBSD. The additional spots in Fig. 6b result from the double diffraction. Similar patterns and the formation mechanism of such double-diffraction spots have been reported in the previous study^12^. The OR between the grain-boundary M_2_C carbide particle (~144 nm) and the surrounding matrix was verified by TEM, as shown in Fig. 6d. The deviation angles from the strict OR were measured to be ~3°, which agrees with the EBSD results (Fig. 5g).

### Hardness

**Figure 7:**
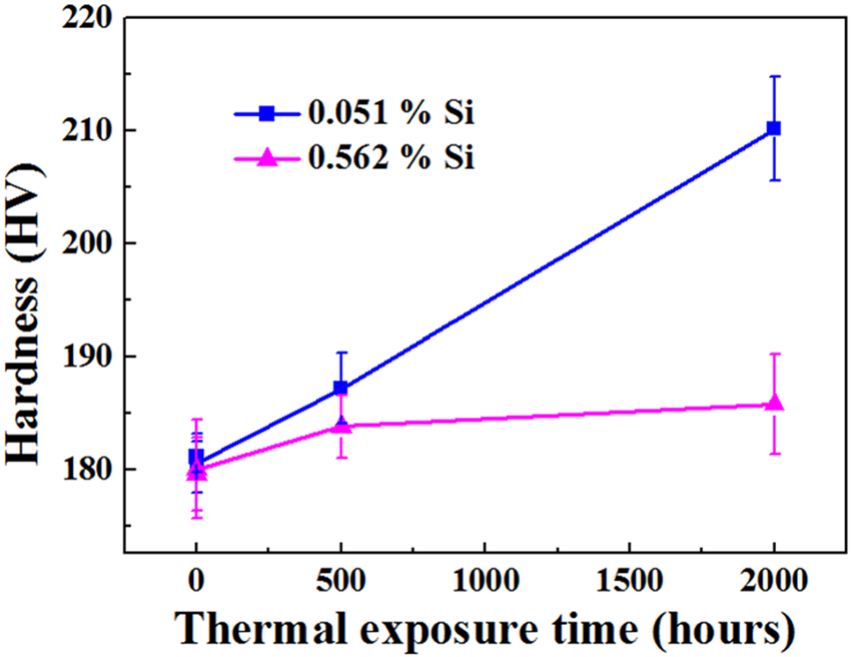
Hardness of the Si-free and Si-containing alloys as a function of the exposure time.

The Vickers hardness of the Si-free and the Si-containing alloys exposed to 650 °C for different times were measured (Fig. 7). No hardening is apparent in the two alloys after thermal exposure for one hour. With increasing thermal exposure, the hardness of the Si-free alloy increases continually, while the hardness of the Si-containing alloy remains almost unchanged.

## Discussion

### Formation of M_2_C and M_6_C carbides at grain boundaries

Adding Si has been proven to suppress the formation of M_2_C carbides and promote the precipitation of M_6_C carbides at the grain boundaries in GH3535 alloy^4^. The critical Si content that completely suppresses the formation of M_2_C carbides was found to be >0.188 wt.%. Thus, the appearance of M_2_C and M_6_C carbides at the grain boundaries in the Si-free and Si-containing alloys, respectively (Figs 1 and 2), is reasonable. In our previous study, M_2_C carbides were found to be instable in the Si-free alloy during materials processing or thermal exposure above 700 °C. When the Si-free alloy was exposed from 700 °C to 800 °C, the M_2_C carbides precipitated preferentially at the grain boundaries during the initial stage and then completely dissolved and transformed into massive, plate-like M_6_C carbides by a separate nucleation and growth process with increasing exposure time^3,4^. The exposure times required for the complete transformation at 700 °C, 750 °C and 800 °C are 500 hours, 200 hours and 100 hours, respectively. In contrast, M_2_C carbides remain after exposure to 650 °C for 2000 hours in the present work. The M_2_C carbides are expected to remain stable at 650 °C or below.

C atoms and carbide-forming elements including Mo and Cr have been widely observed to segregate at grain boundaries before the nucleation of carbides in superalloys^13–17^. Additionally, C and Mo atoms have been proven to diffuse along Ni grain boundaries orders of magnitude faster than through the lattice in the temperature range of carbide formation^18,19^. Thus, M_2_C or M_6_C carbides form preferentially at the grain boundaries rather than in the grain interior (Figs 1 and 2) and exhibit a shorter incubation time for nucleation and stronger coarsening kinetics at the grain boundaries (Fig. 4).

In this study, the M_2_C carbides in the Si-free alloy exhibit more sluggish coarsening behaviour than the M_6_C carbides in the Si-containing alloy at 650 °C. The mean size of M_2_C carbides increases to ~169 nm at grain boundaries (Fig. 4). In the Si-containing alloy, the grain-boundary M_6_C carbides are as large as ~360 nm. On the other hand, the M_2_C carbides in the Si-free alloy exhibit a longer incubation time for nucleation than the M_6_C carbides in the Si-containing alloy (500 hours vs. one hour). The obvious differences in the nucleation and coarsening behaviours between the M_2_C and M_6_C carbides can be related to their compositional characteristics. The coarsening behaviours of precipitates are controlled by the diffusion of the constituent elements from the surrounding matrix. To simplify the analysis, we take pure Ni as an example. The diffusion coefficient for the constituent elements of the M_2_C and M_6_C carbides, such as Mo, Cr and C, are 2.8 * 10^–14^ cm^2^/sec (800 °C)^19^, 6.7 * 10^−12^ cm^2^/sec (650 °C)^20^ and 5.1 * 10^−10^ cm^2^/sec (650 °C)^21^, respectively. The coarsening behaviours of the M_2_C and M_6_C carbides can be deduced to be controlled by the diffusion of Mo in the matrix. Due to the formation of Mo-rich primary M_6_C carbides, only 12 wt.% Mo is present in the matrix of this alloy after the solution treatment^9^. In the M_2_C and M_6_C carbides, the proportions of Mo in metal atoms (wt.%) are ~84 and ~55, which were respectively converted from the atomic ratio values as shown in Figs 1f and 3d. Thus, the enrichment ratios of Mo in the M_2_C and M_6_C carbides are ~7 and ~4.6, respectively. Obviously, more Mo atoms are required for the nucleation and coarsening for M_2_C carbides, which leads to the sluggish nucleation and coarsening behaviours.

### Formation of M_2_C carbide arrays in the Si-free alloy

**Figure 8:**
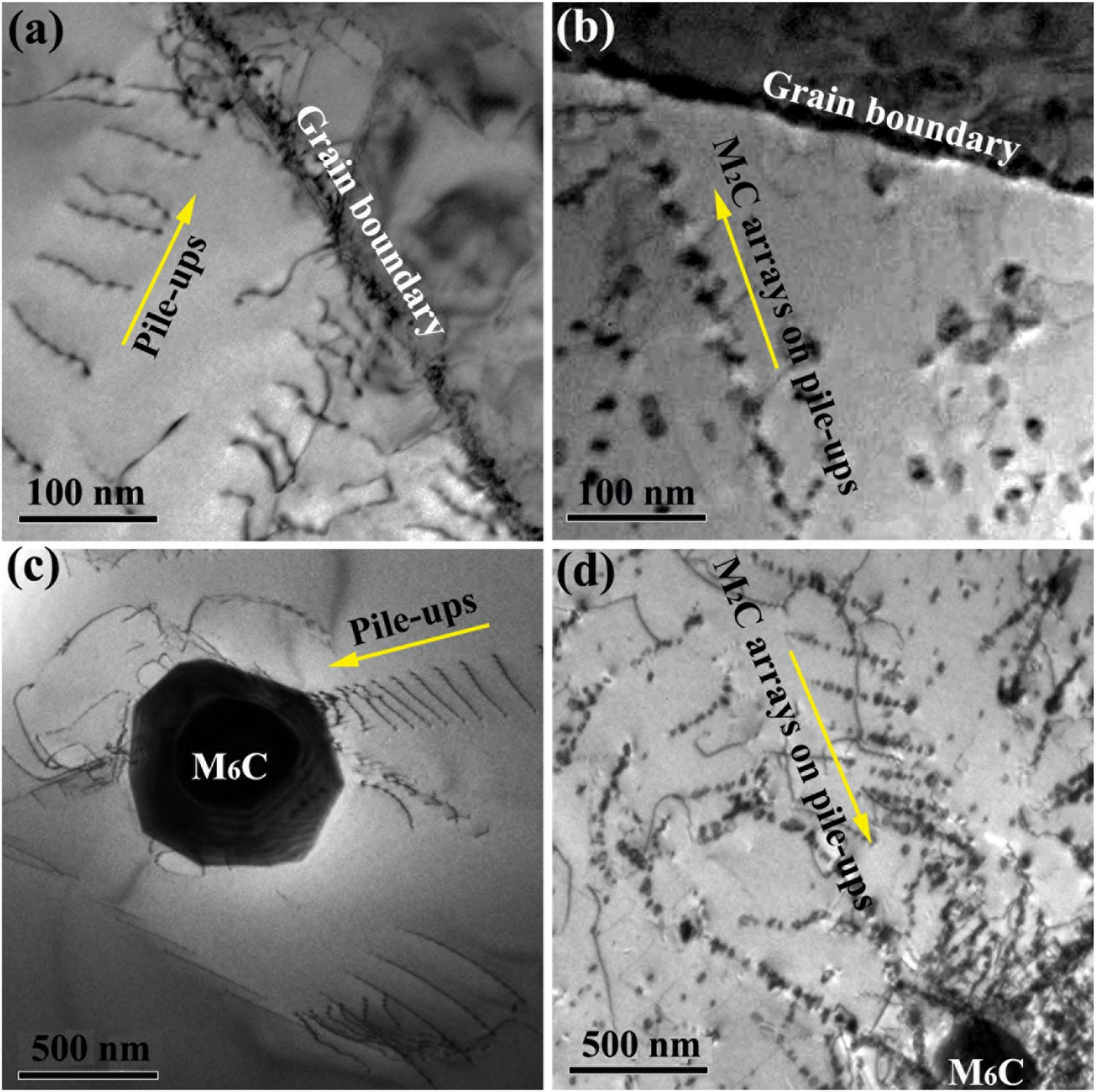
Precipitation of M_2_C carbide arrays induced by pile-ups. **(a)** Pile-ups near grain boundaries in the solution-treated Si-free alloy. **(b)** Pile-up induced M_2_C carbide arrays near the grain boundaries in the Si-free alloy exposed for 2000 hours. **(c)** Pile-ups near the primary M_6_C carbides in the solution-treated Si-free alloy. **(d)** Pile-up induced M_2_C carbide arrays near the primary M_6_C carbides in the Si-free alloy exposed for 2000 hours.

As shown in Figs 2 and 3, the M_2_C carbide arrays grow along the {1 1 1} planes of the matrix. In many alloys, the precipitates along the {1 1 1} planes are always characterized by a sheet-like morphology^22–24^. In one given OR, the precipitates/matrix interfaces tended to be parallel to the {1 1 1} planes of the matrix and exhibited the best atomic correspondence and the lowest interface energy^25^. In the present study, all M_2_C carbides possess a more equiaxed morphology. Peng and Chou^11^ evaluated the effects of OR on the morphology of the M_2_C carbides in the FCC matrix based on Dahmen’s invariant line theory^26^. They found that no growth direction was preferred. The formation of arrays can thus be concluded to be unrelated to the OR. On the other hand, M_2_C carbide arrays are always accompanied by dislocations. As shown in Fig. 8, pile-ups can be observed near grain boundaries (Fig. 8a) and the primary M_6_C carbide particles (Fig. 8c) in the solution-treated Si-free alloy. These pile-ups could be left over from hot rolling or water quenching. After the thermal exposure of 2000 hours, the M_2_C carbide arrays can also be observed at the same sites, and they possess the same morphologies as pile-ups (Fig. 8c vs. d). These facts imply that the preformed dislocations on the {1 1 1} planes (slip planes) of the matrix induced the nucleation of the M_2_C carbides. The dislocation pile-ups near the grain boundaries act as nucleation sites for the initial M_2_C carbide arrays after 500 hours of exposure (Fig. 2d). With increasing exposure time, the Mo and C atoms at the grain boundaries diffuse along the preformed M_2_C carbide arrays to the other dislocations on the slip planes to support the nucleation and growth of new M_2_C carbides in the grain interior (Fig. 3c).

### Formation of PFZs and PEZs in the Si-free alloy

PFZs have been widely reported in Al-based and Ni-based alloys. For Ni-based alloys, the formation mechanism is the competition between intragranular and intergranular precipitations with the same constituent elements^27^. The width of the PFZ thus depends on the competition between the diffusion of the constituent elements towards the intergranular precipitates and the ability of the intragranular precipitates to nucleate and grow in the matrix.

**Figure 9:**
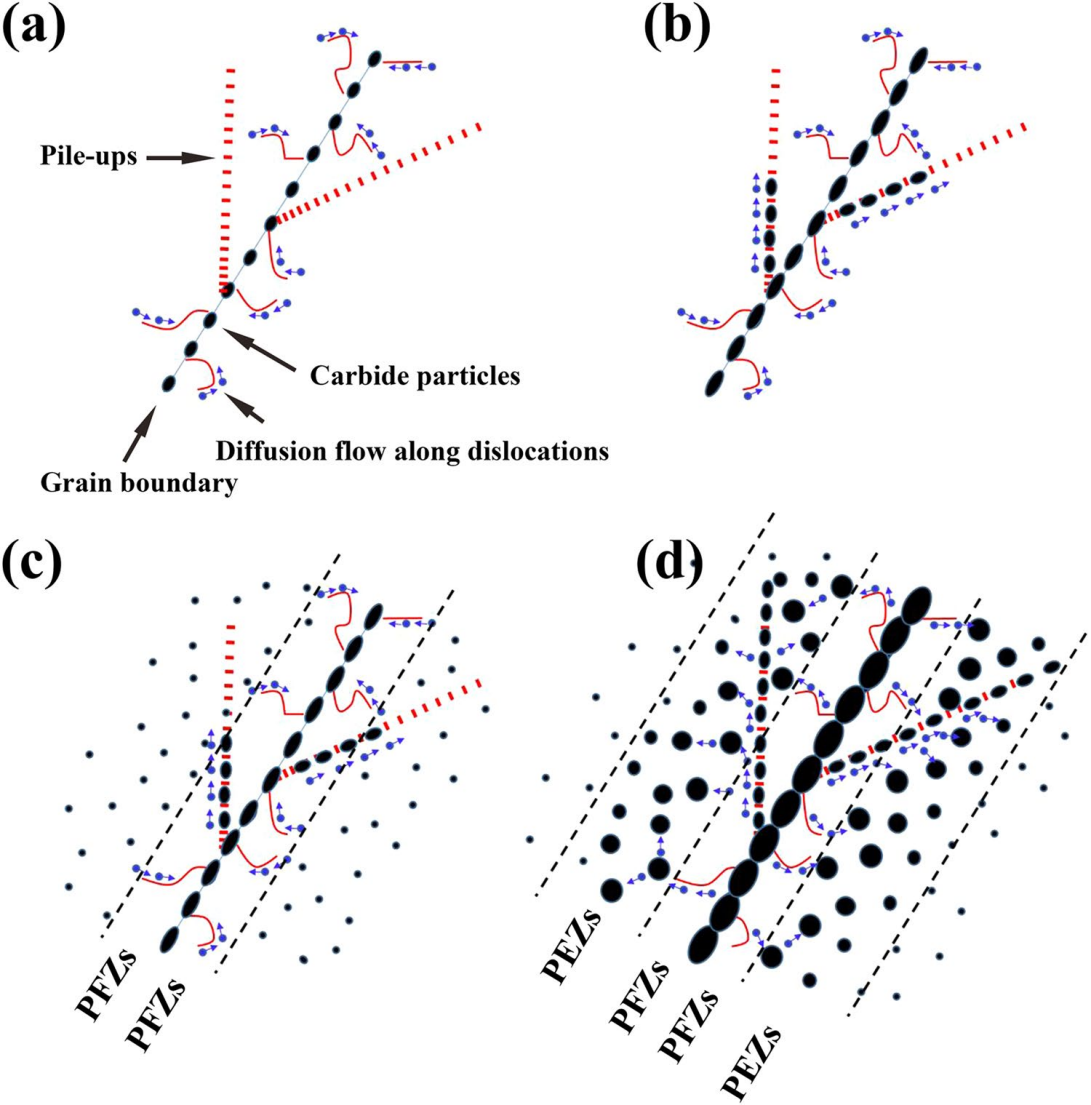
Formation of PFZs and PEZs in the Si-free alloy at different stages of thermal exposure. **(a)** Before 500 hours, **(b)** 500 hours, **(c)** between 500 hours and 2000 hours, and **(d)** 2000 hours.

The formation processes of the PFZs and PEZs in the Si-free alloy are illustrated in Fig. 9. As mentioned above, the segregation and rapid diffusion of Mo and C atoms promote the preferential formation of grain-boundary M_2_C carbides (Fig. 9a) and arrays (Fig. 9b) during the initial stage of exposure. The area adjacent to the grain boundaries becomes depleted of this element, thus hindering the nucleation of new M_2_C carbides in this area. In the grain interior far from the grain boundaries, the elemental distribution and precipitate nucleation are immune to such effects, and M_2_C carbides nucleate at the randomly distributed dislocations and grow (Fig. 9c). Then, the area adjacent to the grain boundaries can be observed to be free of precipitates, i.e., PFZs.

As shown in Figs 2f and 3a, PEZs coexist with the PFZs, which is rarely reported in other systems. After 2000 hours of exposure, the grain-boundary M_2_C carbides become dense (Fig. 3b), which implies that their coarsening kinetics become sluggish. At another border of the PFZs, the M_2_C carbides are still in the initial stage of growth, during which they strongly absorb Mo and C atoms from the surrounding matrix. The difference in the coarsening kinetics drive the Mo and C atoms from the grain boundaries to another border of the PFZs to support the growth of the M_2_C carbides. The preformed arrays and dislocations cross the PFZs and act as diffusion paths (Fig. 9d). Then, the M_2_C carbides at another border of the PFZs coarsen, and new M_2_C carbide particles nucleate in this region to form PEZs.

### Effect of M_2_C carbides on the hardness and post-irradiation properties

As mentioned above, nano-sized M_2_C carbides in the Si-free alloy are more stable in terms of size and OR than the M_6_C carbides in the Si-containing alloy at 650 °C. More importantly, nano-sized M_2_C carbides exist in the matrix in the exposed Si-free alloy, which enhances the hardness (Fig. 7).

Additional valuable results about the post-irradiation properties were reported by Martin and Weir^28^. They found that the post-irradiation creep properties of Si-free alloys (0.015 wt.%) are superior to those of Si-containing ones (0.58 wt.%) when irradiated at 650 °C. This result is meaningful for material selection and design; however, interpretations of this result are lacking. The Si contents of the Si-free and Si-containing alloys in their study and ours are comparable. Thus, we attempt to explain the advantage of the post-irradiation properties of the Si-free alloy at 650 °C by investigating the microstructure.

The high-temperature irradiation damage of Hastelloy N alloy has been manifested as a 10-fold reduction in the creep-rupture life and a 75 to 100% reduction in the fracture strain^29^. The damage mechanism is the formation of helium from a thermal neutron reaction with the residual ^10^B in the alloy^29^. The low solubility was equivalent to a very strong tendency for helium to precipitate into clusters or bubbles at dislocations, precipitates and grain boundaries^30^. At grain boundaries, helium bubbles serve as crack nuclei and cause intergranular fracture. Thus, protecting grain boundaries from helium or blocking crack propagation due to helium bubbles are two solutions to improve the post-irradiation properties of this alloy.

The OR has been proposed to improve the interfacial cohesive force between the carbides and the matrix^31,32^. In the Si-free alloy, finer M_2_C carbides with a certain OR along the grain boundaries hinder crack propagation along the grain boundaries and increase the resistance to intergranular fracture. On the other hand, fine precipitates in the grain interior can provide enough phase interfaces to trap the helium rather than allowing it to be swept into the grain boundaries. In a typical case, fine MC carbides were found to improve the post-irradiation properties of the Fe-25Ni-15Cr alloy^33,34^. In this study, the mean size of the M_2_C carbides in arrays increases slightly from ~40 nm to ~53 nm after 2000 hours of exposure, and the randomly distributed particles in the grain interior possess a mean size of ~26 nm (Fig. 4b). In contrast, no secondary precipitates except the thick primary M_6_C carbides can be observed in the grain interior in the exposed Si-containing alloy. Less helium is expected to sweep into the grain boundaries in the Si-free alloy due to the M_2_C carbides. In particular, the high density of M_2_C carbides in PEFs should be better able to trap helium and protect the grain boundaries from helium.

In summary, nano-sized M_2_C carbides can be observed in the grain interior (as arrays or randomly distributed particles), in PEZs and at grain boundaries and in the Si-free alloy after being exposed to 650 °C. The morphology and OR of nano-sized M_2_C carbides enable them to inhibit crack propagation and trap helium, which are expected to result in better post-irradiation properties, as reported by Martin and Weir^28^. Additionally, our results indicate that the morphology and OR of the M_2_C carbides in the grain interior exhibit high stability after long-term thermal exposure at 650 °C. More efforts are needed to evaluate this alloy’s potential for low-temperature components at 650 °C.

## Conclusions

1. After long-term thermal exposure at 650 °C, M_2_C carbides can be observed in the grain interior (as arrays or randomly distributed particles) and at grain boundaries and in adjacent PEZs. The M_2_C carbides mainly contain Mo, Cr and a small number of metallic Ni atoms.
2. The M_2_C carbides were found to possess a special OR with the matrix γ phase, which can be stated as ${(0001)}_{c}//{(111)}_{\gamma }$, ${(11\bar{2}0)}_{c}//{(1\bar{1}0)}_{\gamma }$ and ${(\bar{1}100)}_{c}//{(11\bar{2})}_{\gamma }$.
3. The morphology and OR of the M_2_C carbides in the grain interior are more stable than those at the grain boundaries after long-term thermal exposure at 650 °C.
4. The better post-irradiation properties of the Si-free alloy, which were reported previously, are related to the nano-sized M_2_C carbides and their specific OR with the matrix.

## Methods

### Sample preparation

Si-free and Si-containing GH3535 alloy ingots were prepared by vacuum induction melting (VIM) a mixture of pure Ni (99.9%), Mo (99.9%), Cr (99.5%), Fe (99.9%), Mn (99.9%), Si (99.9%) and graphite (99.9%). The ingots were then hot rolled into bars with a 20 mm diameter. All specimens were cut from the bars and then solution heated at 1177 °C for 0.5 hour, followed by water quenching. The prepared specimens were then thermally exposed to 650 °C for 1, 500 and 2000 hours, respectively. The chemical compositions (wt.%) of the two alloys were detected using an optical emission spectrometer (SPECTROMAXx06) and a carbon–sulfur spectrometer (LECO CS844), and the results are shown in Table 1. The trace amount of Si in the Si-free alloy was determined to be as low as 0.051 wt.%.

**Table 1:** Composition of the Si-free and Si-containing GH3535 alloy (wt.%).

Element	C	Si	Mn	Cr	Fe	Mo	Ni
Si-free	0.054	0.051	0.525	6.96	4.00	16.15	Bal.
Si-containing	0.057	0.562	0.530	6.88	4.01	16.11	Bal.

### Characterization

Metallographic samples were prepared using standard metallographic techniques, and the polished specimens were etched with a mixture solution (3 g CuSO_4_ + 10 ml H_2_SO_4_ + 40 ml HCl + 50 ml water) for 30 seconds. The microstructures were examined using a SEM (Zeiss Merlin Compact). A vibration-assisted final polishing was carried out for the EBSD specimens to improve the surface finish and to remove the damaged layer caused by mechanical polishing. The ORs between the precipitates and the matrix were investigated using EBSD, which was performed on the SEM equipped with an Oxford Instruments AZtec system. An accelerating voltage of 20 kV was used for these operations. TEM (Tecnai G2 F20 S-TWIN) was used for the phase identifications, microstructural observations, and compositional analyses. The TEM specimens were prepared by electrochemical polishing with a solution of 5% perchloric acid and 95% alcohol at approximately 243 K.

The surface hardness was measured using a Vickers indenter in a Zwick hardness tester (ZHVμ-S) under a load of 2000 gf. At least 10 test points were measured for each sample. The sizes of the indentations range from 130 μm to 150 μm to sufficiently reflect the effect of the microstructural evolution on the hardness.
